# Neuroinflammation and pathways that contribute to tourette syndrome

**DOI:** 10.1186/s13052-025-01874-3

**Published:** 2025-02-28

**Authors:** Xinnan Wu, Juanjuan Hao, Keyu Jiang, Min Wu, Xin Zhao, Xin Zhang

**Affiliations:** 1 Xin Hua Hospital, Shanghai Jiao Tong University School of Medicine, Shanghai, China; 2 School of Medicine, Shaoxing University, Shaoxing, China

**Keywords:** Tourette syndrome, Immune response, Neuroinflammation, Bacteria, Virus, Allergen

## Abstract

Tourette syndrome (TS), a neurological and psychological disease, typically exhibit motor and phonic tics. The pathophysiology of TS remains controversial. Currently, the recognized pathogenesis of TS is the imbalance of neurotransmitters, involving abnormality of the cortex-striatum-thalamus-cortex circuit. Recently, clinical researches demonstrate that triggers such as infection and allergic reaction could lead to the onset or exacerbation of tic symptoms. Current studies have also suggested that neural-immune crosstalk caused by inflammation is also associated with TS, potentially leading to the occurrence of tics by inducing neurotransmitter abnormalities. Herein, we review inflammation-related factors contributing to the occurrence of TS as well as the mechanisms by which immune-inflammatory pathways mediate the onset of TS. This aims to clarify the pathogenesis of TS and provide a theoretical basis for the treatment of TS.

**Supplementary Information:**

The online version contains supplementary material available at 10.1186/s13052-025-01874-3.

## Introduction

Tourette syndrome (TS) is a neurodevelopmental disorder that begins in childhood and is characterized by numerous involuntary motor and vocal tics lasting over a year [1]. Approximately 0.3–1% of the population is affected by TS [2]. TS frequently co-occurs with attention deficit/hyperactivity disorder, obsessive-compulsive disorder (OCD), and other psychological problems [3, 4]. TS can affect daily life, severely affecting physical and mental health, causing a decline in academic performance, and even leading to social impairment.

Research on the pathogenesis of TS has involved the investigation of genetics; neurotransmitters; and environmental, immunological, and other factors [5–7]. Among these, an imbalance in neurotransmitter levels is one of the most recognized pathogenic mechanisms. Studies have shown that tics may result from the loss of inhibition of motor cortical neurons and dysfunction of the cortex-striatum‐thalamus‐cortex (CSTC) circuit [8–10]. As the main neurotransmitters of the CSTC circuit, dopamine (DA) and glutamate (Glu) have been shown to be associated with the onset of TS [11–13].

In recent years, increasing evidence has indicated that neuroinflammation is mediated by infections or allergic reactions during the pathogenesis of TS and other neuropsychiatric disorders [14, 15]. Studies have demonstrated that there is a subset of patients with TS in whom tic symptoms are induced by infections or allergic reactions [16, 17]. In addition, most patients with TS present exacerbated symptoms after pathogenic infections or allergic reactions [18, 19]. Some clinical and basic studies have focused on the pathogenesis of TS resulting from infections or allergic reactions.

How these abovementioned factors induce the development of TS is not clear. Therefore, here, we briefly review the mechanisms by which inflammatory responses triggered by bacteria, viruses, and allergens mediate an imbalance in neurotransmitters that leads to the onset of TS.

This article provides a narrative review focusing on inflammation-related factors contributing to the occurrence of TS and the mechanisms by which immune-inflammatory pathways mediate tic onset. A systematic literature search was conducted in databases such as PubMed and Web of Science, including studies from the past two decades on the relationship between TS and immune-inflammatory pathways. The following keywords were used: ‘Tourette syndrome’, ‘inflammation’, ‘immune’, ‘microglia’, ‘neural-immune crosstalk’, ‘anti-neuronal antibodies’, and ‘infection’. This review aims to integrate the current evidence on the immune-inflammatory mechanisms underlying TS pathogenesis.

## Etiology

### Bacterial infection

Among the multiple bacterial strains, streptococcal infection is considered a recognized trigger of TS. Studies have indicated that TS is likely related to prior streptococcal infections [20, 21]. Among the numerous types of streptococci, Group A streptococci (GAS) are the most closely associated with TS [22–25]. Group A beta-hemolytic streptococci (GABHS), the most common pathogenic strain of GAS in children, is associated with the onset of TS [26]. Studies have defined a separate category of TS known as pediatric autoimmune neuropsychiatric disorders associated with streptococcal infections (PANDAS), based on the hypothesis that autoimmunity induces neuropsychiatric symptoms [18]. Among children diagnosed with PANDAS, exacerbations and relapses of tic symptoms were linked to GABHS infections [18, 19]. If the PANDAS theory is confirmed, it would support the idea that tic disorders are related to streptococcal infections.

*Staphylococcus aureus* can also cause worsening of tic symptoms in patients with TS, and its pathogenesis is related to the immune response during bacterial clearance [27]. Lung infections with bacteria, such as *Pseudomonas aeruginosa*, have been reported to cause the release of systemic cytokines and neuroinflammation, leading to behavioral changes in patients with TS [28]. Therefore, other bacteria that are expected to trigger TS are expected to be discovered in the future.

### Viruses

The correlation between TS and viruses, such as enterovirus (EV), human immunodeficiency virus, herpes simplex virus, varicella zoster virus, cytomegalovirus, coxsackievirus B, and severe acute respiratory syndrome coronavirus 2, has been reported in multiple studies [29–34]. During the COVID-19 pandemic, tic-like behaviors emerged in young people, indicating that COVID-19 may also be associated with the occurrence of TS [31]. SARS-CoV-2, as a model of infection which could lead to neuroinflammation, may also play a significant role in triggering or exacerbating TS [35].

### Other pathogens

Other pathogens associated with TS have also been reported, including *Chlamydia trachomatis*, *Chlamydia pneumoniae*, *Mycoplasma pneumoniae*, *Toxoplasma gondii*, and *Borrelia burgdorferi*. *B. burgdorferi* and *M. pneumoniae* have been reported to induce tic exacerbations [25, 36–40].

### Allergic reactions

Many studies have suggested that allergies may cause the onset of TS [41]. The prevalence of allergic diseases is higher in TS patients than in the general population [17, 42, 43]. A meta-analysis reported that tic syndrome was related to allergic diseases, such as allergic rhinitis, eczema, asthma, food allergy, and allergic conjunctivitis; however, it was not related to urticaria, atopic dermatitis, or drug allergy [44].

Children with TS were found to have positive skin tests and higher serum IgE levels, mainly against inhalation allergens, such as dust mite combinations, indicating the occurrence of allergic reactions [42, 45]. When allergens are encountered, plasma cells produce IgE, and histamine is released when IgE reacts with the allergen. High IgE levels are thought to result in allergic reactions as well as excessive release of inflammatory cytokines [45], which would damage striatal dopaminergic neurons, causing the disruption of dopaminergic signals, thereby causing tic disorders.

## Pathogenesis

### Inflammatory factors

Pathogens cause neurotransmitter imbalances through the following mechanisms. They can damage neurons by activating T cells to produce inflammatory factors or B cells to produce anti-neuronal antibodies. Both these factors may lead to the onset of TS. A lack of Treg cells was found in patients with TS, which enhanced the elimination of the infectious pathogen. Pathogenic infections can lead to hyperactivation of the peripheral immune system and release of excessive inflammatory factors. These inflammatory factors may lead to the dysfunction of neural-immune crosstalk, which may cause an imbalance in neurotransmitters, such as DA and Glu, which can lead to tics. Pathogenic infections can also induce the production of anti-neuronal antibodies by activating B cells. Anti-neuronal antibodies interact with neuronal surface antigens and activate microglia, leading to the damage of dopaminergic neurons, ultimately resulting in TS.

#### Production of inflammatory factors mediated by activation of the peripheral immune system

Impaired immune tolerance to self-antigens in patients with TS might result from a deficiency of Treg cells in TS patients [46], which may reduce the ability to suppress self-reactive T lymphocytes. Subsequently, an overactivated autoimmune response enhances the elimination of infectious agents. When pathogens infect the body, overactivation of the autoimmune response leads to the massive release of inflammatory factors. Peripheral inflammatory factors can increase the permeability of the blood-brain barrier (BBB), possibly inducing a neurotransmitter imbalance by affecting microglia or astrocytes, which in turn lead to the onset of TS.

##### Lack of treg cells in patients with TS

A lack of Treg cells has been found in patients with TS, which might result in a lower ability to suppress self-reactive T lymphocytes, leading to impaired immune tolerance to self-antigens [46]. Self-reactive T lymphocytes play a role in defending against pathogenic infections and continue to be present in the peripheral immune cell repertoire [46]. Immune tolerance targets self-antigens and is maintained through various suppressive mechanisms. CD4(+) CD25(+) Treg cells, which inhibit self-reactive T lymphocyte responses to foreign antigens, can mediate peripheral tolerance to self-antigens [47]. The depletion of Treg cells increases the number of CD8 + T cells, enhancing the elimination of infectious pathogens [48]. In some cases, self-reactive lymphocytes can cause damage to the host [46].

##### Over activation of the peripheral immune system

Overactivation of the peripheral immune system has also been observed in patients with TS. Researchers have found an increase in the number of natural killer (NK) and CD8 + T cells, a reduction in CD4 + T cells, and a decrease in the CD4+/CD8 + ratio in patients [15, 49]. One study reported higher plasma IL-12 levels in patients [50, 51]. IL-12 has the ability to drive CD4 + T-cell differentiation into helper T (Th) cells and activate NK cells, indicating that the peripheral immune system of patients with TS is over-activated [52]. Another study indicated an increase in CD95 + Th cells in patients with TS, demonstrating that patients with TS present a hyperreactive immune state [53]. When CD95 (Fas) is activated, it induces cellular apoptosis to remove activated peripheral T cells through its interaction with the Fas ligand, which suggests an increase in peripheral immune activity [54].

##### Release of inflammatory factors

Pathogenic infections may contribute to the onset of TS, which is mediated by host T-cell immunity. Bacteria share epitopes with human self-antigens. When pathogens infect the human body, autoreactive T lymphocytes are activated, resulting in the development of autoimmunity and the inhibition of suppressive mechanisms [46, 55]. Subsequently, the suppressive mechanisms of Treg cells are overturned, and immune tolerance to self-antigens may be impaired, resulting in massive release of pro-inflammatory cytokines [56]. Higher serum levels of soluble CD14 were detected in patients with TS and bacterial infections [57]. Soluble CD14 stimulates the production of inflammatory cytokines that may increase bacterial resistance [58]. There are also studies showing that viruses stimulate the release of inflammatory factors, such as IL-6 and TNFα, in serum [59]. Previous studies have reported increased serum levels of pro-inflammatory cytokines, such as IL-6, TNFα, IFN-γ, IL-17, IL-12p70, and IL-1β in patients with TS, as well as IL-2 in those comorbid with OCD [15, 50, 51, 60, 61]. Pro-inflammatory cytokines in the serum may cross the BBB and affect microglia and astrocytes in the brain, inducing neurotransmitter abnormalities, which in turn, may lead to the development of TS. Hence, we speculated that pathogenic infections may result in a hyperreactive immune state in the human body, which may induce the onset of TS.

#### Dysfunction of neural-immune crosstalk

Peripheral inflammatory factors can increase BBB permeability, allowing them to cross the BBB. These inflammatory factors may lead to the dysfunction of neural-immune crosstalk through the activation of microglia or other pathways, potentially leading to an imbalance in neurotransmitters and contributing to the onset of TS.

##### The activation of microglia caused by inflammatory factors

IFN-γ, TNF-a, and IL-6 have been shown to be efficient at crossing the BBB, entering the cerebral vasculature or brain tissue [62–64]. The levels of IL-6 and TNF-α are upregulated in the brain tissue of rats with TS [65]. IL-6 and TNF-α damage the brain in different ways. TNF-α indirectly enhances the production of potentially neurotoxic metabolites, to disrupt brain development by adjusting neurotransmitter metabolism [66]. Microglia in the brain may be activated by pro-inflammatory cytokines from the serum, which may lead to an increase in neuronal excitability and the release of more inflammatory factors in the brain [64]. Recent studies have suggested that microglia play an important role in neuroinflammation, which is associated with tic disorders.

The activation of microglia in the brain mainly results from higher levels of chemokine ligand 5 (CCL5) in the blood, upregulated genes related to immunity, and a lack of histamine (HA). The following section provides a detailed description of the three pathways involved in microglial cell activation.

(1) Higher blood CCL5 levels: Pathogenic infections induce the overactivation of T lymphocytes [55]. CCL5, released by immune cells, such as T lymphocytes and macrophages, plays an important role in recruiting leukocytes to inflammatory sites. A previous study reported higher blood CCL5 levels in patients with TS [67]. CCL5 enters the brain by crossing the BBB and interacting with its receptors, C-C chemokine receptor type 5 (CCR5) and C-C chemokine receptor type 1 (CCR1). Neurological impairments may result from CCL5-CCR1-mediated microglial activation through the CCR1/TPR1/ERK1/2 signaling pathway [68]. CCL5 interacts with CCR5. The activation of CCR5 can promote neuronal pyroptosis via the CCR5/PKA/CREB/NLRP1 signaling pathway, which may cause neuronal impairment and induce the onset of tics [69].

(2) Upregulated genes related to immunity: One study reported upregulated hub genes, including intercellular adhesion molecule 1, C-C motif chemokine ligand 2, heme oxygenase 1, MYC proto-oncogene, and suppressor of cytokine signaling 3, in patients with TS [70]. Studies have found that the hub genes upregulated in TS are commonly related to immune and inflammatory pathways that involve the interleukin and interferon signalling pathways [70]. Another study also reported that the upregulated genes in the caudate and putamen of individuals are mostly immune-related genes, which are related to the activation of microglia and can induce the excessive release of inflammatory factors [71].

(3) The lack of HA: HA deficiency promotes the release of the inflammatory factors like IL-1β [72], while making microglia more susceptible to inflammatory challenge and promoting microglia M1 hyperpolarization. A lack of HA can also promote microglial activation, which has been proposed as a potential cause of TS. HA acts as an anti-inflammatory substance to inhibit lipopolysachharide (LPS)-stimulated exacerbated microglial responses via histamine H4 receptor activation and inhibit the release of IL-1β [72]. HA also regulates microglial functions [73]. Histidine decarboxylase (Hdc), an enzyme essential for HA synthesis, plays an important role in TS [72, 74]. A decreased number of IGF-1-positive microglial cells were found in Hdc-knockout mice [73]. IGF-1-positive microglia protect the brain. However, this protective function is weakened when the number of IGF-1-positive microglia is reduced. Consequently, HA deficiency renders microglial cells more vulnerable to inflammatory challenges mediated by LPS. Subsequently, microglia produce inflammatory factors that damage neurons and may lead to tics.

**Fig. 1:**
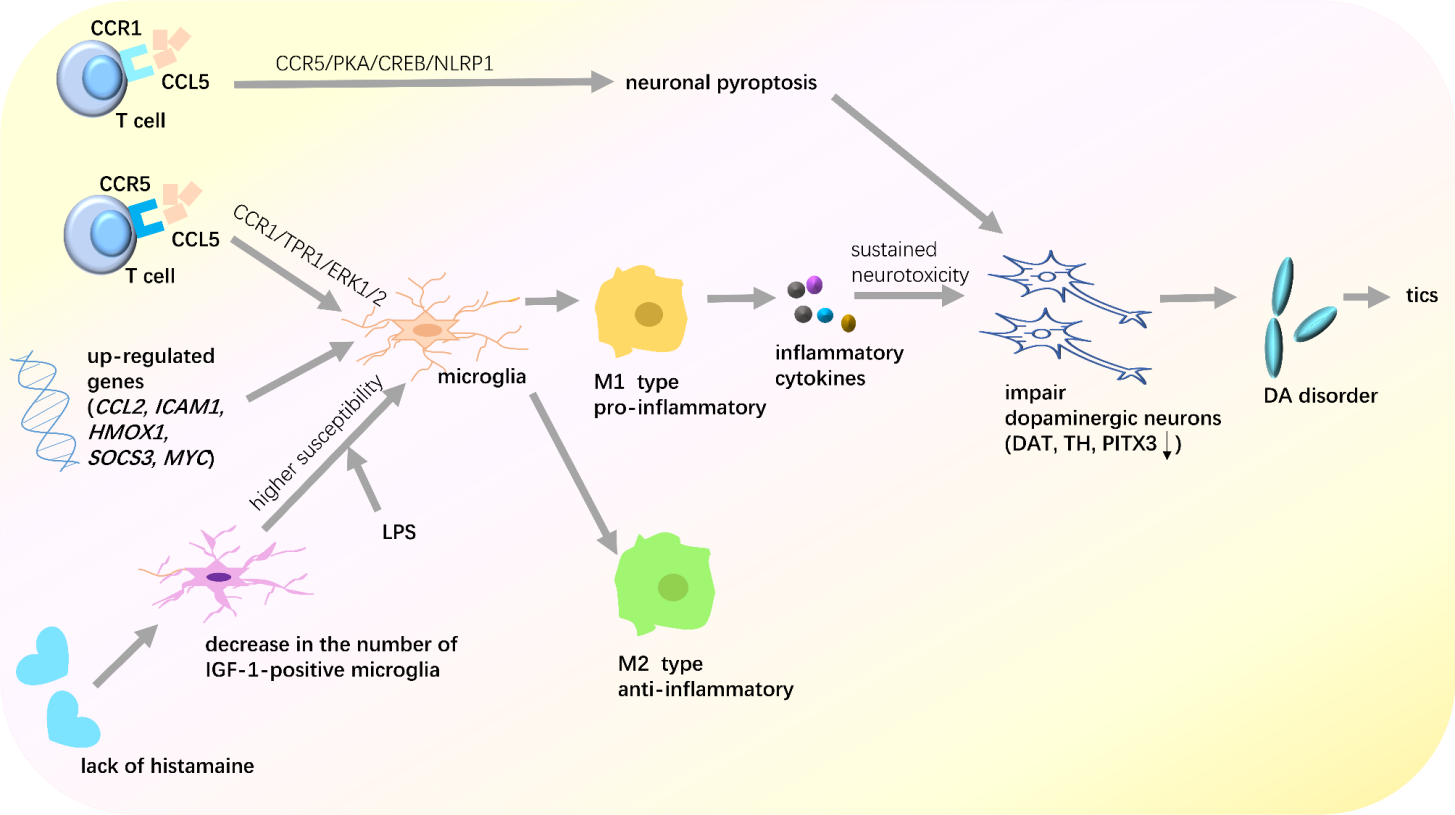
Overview of microglia M1 polarization–mediated tic onset. The activation of microglia mainly results from three processes, including increased chemokine ligand 5 (CCL5) levels in the blood, up-regulated immune-related genes, and a lack of histamine (HA). CCL5 in the blood may enter the brain and interact with its receptors, C-C chemokine receptor type 5 (CCR5) and C-C chemokine receptor type 1 (CCR1). The activation of CCR5 promotes neuronal pyroptosis through the CCR5/PKA/CREB/NLRP1 signaling pathway. The activation of CCR1 may result in neurological impairments through the CCR1/TPR1/ERK1/2 signaling pathway. Both of these pathways may cause neuronal impairment and induce the onset of tics. Up-regulated genes related to immunity and inflammation, including C-C motif chemokine ligand 2 (CCL2), intercellular adhesion molecule 1 (ICAM1), heme oxygenase 1 (HMOX1), MYC proto-oncogene (MYC), and suppressor of cytokine signaling 3 (SOCS3), are related to the activation of microglia. The lack of HA may lead to a decrease in the number of IGF-1-positive microglia cells, which have the function of protecting the brain. As a result, HA deficiency increases the susceptibility of microglial cells to inflammation triggered by lipopolysaccharide (LPS). M1-type microglia are known as pro-inflammatory microglia. Microglia M1 hyperpolarization may lead to an increase in inflammatory cytokine levels and sustained neurotoxicity. Striatal dopaminergic neurons are then impaired, which may cause tic disorders, and tics may occur subsequently

Microglia are divided into two types, namely M1-type and M2-type [75]. M1-type microglia, which are the classical pro-inflammatory type of microglia, release inflammatory factors and induce neuroinflammatory and neurotoxic responses [76]. All three approaches mentioned above can induce microglial M1 polarization. One study found that microglial M1 polarization may cause inflammatory impairment in striatal dopaminergic neurons [13]. Subsequently, dopaminergic signaling is impaired, which may lead to the development of tics. These results indicate that the cooperation between dopamine dysregulation and immune dysfunction may be the underlying cause of TS (Fig. 1).

##### Other mechanisms of neural-immune crosstalk dysfunction caused by inflammatory factors

Inflammatory factors contribute to tics through other pathways. Inflammatory factors may affect astrocyte-neuron metabolic coupling, or lead to dysfunction of the gut-brain axis, or may have an effect on the kynurenine pathway (KP) [29, 76–78]. All of these effects may disrupt the neurotransmitter balance in the brain, which may lead to TS.

Astrocyte-neuron metabolic coupling could induce TS due to neuroimmune interactions. Astrocytes exhibit a neurotoxic phenotype in response to immunological and inflammatory conditions [76]. Dysfunction of astrocyte glutamate transporter 1 results in its loss of function in the regulation of corticostriatal synapses and leads to pathological repetitive behaviors [12, 79, 80].

Dysfunction of the gut-brain axis can influence nervous system development, which may induce or aggravate TS [77]. Streptococcal infections have the potential to modify the composition of the gut microbiota in the human body [81, 82]. Differences in the composition of the gut microbiota may influence the brain-gut axis and alter neurotransmitter levels, potentially contributing to TS symptoms [83]. A higher abundance of *Prevotella* has been reported to increase the levels of inflammatory factors in the gut [84], which may cross the BBB and impair the nervous system through the inflammasome signaling pathway [85]. Increased levels of *Odoribacter* may result in a greater release of dopamine, leading to tics [81]. γ-aminobutyric acid (GABA), an inhibitory neurotransmitter, is reported to be produced by *Bifidobacterium* [86]. *Bifidobacterium* deficiency leads to a decrease in GABA levels in the primary sensorimotor cortex in patients with TS, probably causing allergies as well, thereby contributing to a higher risk of developing motor and vocal tics [87].

Neurotrophic infectious agents can activate tryptophan catabolism and increase the levels of pro-inflammatory cytokines, both of which may affect the neurotransmitter balance in the brain through the KP [29, 78]. Tryptophan is degraded to kynurenine through the KP, which is the main pathway for tryptophan breakdown [78]. These degradation products can act as N-methyl-D-aspartate (NMDA)-receptor antagonists, and have been reported to induce glutamatergic hypofunction and regulate neurotransmitters [88]. Kynurenic acid, the only known endogenous NMDA antagonist, blocks nicotinergic acetylcholine receptors at low doses [88]. The KP may contribute to glutamatergic hypofunction and block nicotinergic acetylcholine receptors, leading to tic disorders.

### Anti-neuronal antibodies

Anti-neuronal and antinuclear antibodies have been found in the serum of patients with neuropsychiatric symptoms, such as TS [89–91]. Pathogenic infections, particularly streptococcal infections, may induce the emergence of anti-neuronal antibodies [92–94],. Streptococcal infections are thought to be associated with ABGA, as well as with TS [22, 27, 95, 96].

Anti-neuronal antibodies have been considered to cross-react with streptococci and antigens in the basal ganglia. The pathogenesis of TS-associated antibodies is speculated to involve cross-reactions between anti-neuronal antibodies and the basal ganglia. Researchers have proposed a mechanism underlying the immune response against streptococcal infections. The GAS cell epitope is similar to lysoganglioside-GM1 and neuronal glycolytic enzymes (NGEs) [97]. One study confirmed that antibodies against lysoganglioside-GM1 or pyruvate kinase (PK), a type of NGE, can react with the GAS cell epitope N-acetyl-beta-d-glucosamine [98, 99]. Therefore, when GAS infects the body, the anti-streptococcal antibodies produced react with neuronal surface antigens. We refer to these antibodies as anti-neuronal antibodies.

The cross-reactivity between IgG antibodies in serum from children with TS and brain tissue has mainly been observed in the CA3 subfields of the hippocampus, the basal ganglia, the cerebellum, and the dentate gyrus (DG) [100]. A few special neuronal surface antigens, such as dopamine-1 receptor (D1R), dopamine-2 receptor (D2R), tubulin, lysoganglioside-GM1, NGE, hyperpolarization-activated cyclic nucleotide channel 4 (HCN4), contactin-associated protein-like 2, the N-methyl-D-aspartate receptor (NMDAR), leucine-rich glioma-inactivated protein 1, the α-amino-3-hydroxy-5-methyl-4-isoxazolepropionic acid receptor, and the γ-aminobutyric acid receptor-A/ the γ-aminobutyric acid receptor-B, have been shown to have more potential to bind with antibodies in patients with TS and related neuropsychiatric disorders [97, 98, 100–112],.

With GAS invading the human body, individuals generate antibodies that recognize specific neuronal surface antigens within the striatum; subsequently, the cross-reactivity of antibodies with the epitopes of the neuronal cells induces the impairment of neuronal function, such as brain reward circuits, ultimately causing tic disorders and other neuropsychiatric damage, which may explain the pathogenesis of TS [91, 113]. Specific neuronal surface antigens that react with antibodies in patients with TS remain ambiguous. Therefore, the priority is to identify new autoantibodies against the neuronal surface antigens [114].

### Signaling pathways involving neural-immune crosstalk

Previous studies have shown that some signaling pathways are involved in TS-mediated neuroinflammation (Table 1). The Ca(2+)/calmodulin-dependent protein kinase II (CaMKII) signaling pathway, JAK-STAT pathway, and NF-κB pathway are crucial pathways involved in the processes of neuroinflammation resulting from the activation of microglia [115].

**Table 1:** Signaling pathways associated with TS mediated by neuroinflammation

Reference	Pathway	Function	Method
[116] (Wu et al., 2023)	CaMKII/Drp1/ROS/NF-κB	Microglia activation	LPS-stimulated BV2 microglial cells
[117] (Huang et al., 2008)	JAK2/STAT3	Mediating microglia activation and dopaminergic neuron degeneration	Thrombin-stimulated rat primary microglia
[118] (Wu et al., 2022)	JAK2/STAT3/p65	Mediating neuroinflammation	LPS-stimulated mouse hippocampal CA1 region and BV2 cells
[119] (Kim et al., 2006)	TLR4/STAT3	Inducing ICAM-1 expression, mediating microglia activation, pro-inflammatory actions	LPS-stimulated mice lacking functional TLR4
[120] (Zeng et al., 2014)	Akt/IκB/ NF-κB	Mediating neuroinflammation	LPS-stimulated BV2 microglial cells
[121] (Kang et al., 2012)	PI3K/Akt/ NF-κB	Inducing pro-inflammatory mediators, NO, PGE(2) and TNF-α, and their regulatory genes	LPS-stimulated BV2 microglial cells
[122] (Hongyan et al., 2017a)	PI3K/Akt/ NF-κB	Increasing the levels of inflammatory cytokines, such as IL-6, IL-1β, and TNF-α, in the serum and striatum of rats	DOI-induced TS model in rats
[123] (Hongyan et al., 2017b)	TLR/MyD88/NF-κB	Increasing the levels of inflammatory cytokines, such as IL-6, IL-1β, and TNF-α, in the serum and striatum of rats	DOI-induced TS model in rats, LPS-stimulated rats
[124] (Long et al., 2019b)	BDNF/NF-κB	Decreasing the BDNF-mediated increase in NF-κB levels; increasing IL-6, IL-1β, and TNF-α levels in the serum, striatum, and cell supernatant of rats with TS	DOI-induced BV2 cells; DOI-induced TS model in rats
[125] (Xu et al., 2017)	BDNF/TrkB/ MyD88/NF-κB	Increasing TrkB expression levels, activating downstream PI3K/AKT signaling after BDNF pretreatment; inhibiting the MyD88/NF-κB signaling pathway; promoting the inflammatory response and hippocampal apoptosis	Pretreatment with exogenous BDNF or the TrkB inhibitor; intracisternal infection with live *Streptococcus pneumoniae*
[126] (Long et al., 2019a)	Nrf-2/HO‐1/HMGB1/NF‐кB	Mediating neuroinflammation	IPN-induced TS model in rats
[127] (Chunhui et al., 2017)	EGF/EGFR/Nrf-2/HO‐1/ NF‐кB	Mediating inflammatory and oxidative injury	osteoblast cells
[128] (Haddad, 2005)	NMDAR/ MAPK/CREB	Regulating the levels of amino acid neurotransmitters; mediating the activation of microglia	DOI-induced TS model in rats; LPS-stimulated BV2 microglial cells
[129] (Hildonen et al., 2021)	PI3K/AKT/ mTOR	Affecting neuronal growth and proliferation; affecting the release of dopamine	An exploratory analysis of the genome-wide DNA methylation patterns in whole-blood samples of 16 monozygotic twin pairs with TS

Abbreviations: Akt: protein kinase B; BDNF: brain-derived neurotrophic factor; CaMKII: calcium-calmodulin dependent protein kinase II; CREB: cAMP-response element binding protein; DOI: 2,5-dimethoxy-4-iodoamphetamine; Drp1: dynamin-related protein 1; EGF: epidermal growth factor; EGFR: estimated glomerular filtration rate; HMGB1: high-mobility group protein B1; HO-1: heme oxygenase-1; ICAM-1: intercellular adhesion molecule-1; IL-1β: interleukin-1β; IL-6: interleukin-6;

IPN: 3,3’-iminodipropionitrile; IκB: NF-κB inhibitory protein; JAK2: Janus kinase 2; LPS: lipopolysaccharide; MAPK: mitogen-activated protein kinase; mTOR: mammalian target of rapamycin; MyD88: myeloid differentiation primary response gene 88; NF-κB: Nuclear factor-kappa B; NMDAR: N-methyl-D-aspartate; NO: nitric oxide; Nrf‐2: nuclear factor erythroid 2-related factor 2; PGE(2): prostaglandin E2; PI3K: phosphoinositide 3-kinase; ROS: reactive oxygen species; STAT3: signal transducer and activator of transcription 3; TLR4: Toll-like receptor 4; TNF-α: tumor necrosis factor-α; TrkB: tropomyosin-receptor kinase; TS: Tourette syndrome

#### CaMKII signaling pathway

The activation of calcium-calmodulin-dependent protein kinase II (CaMKII) has been linked to movement disorders, such as TS [99, 104, 107, 130]. CaMKII activation can be induced by anti-neuronal antibodies or NMDAR. CaMKII activation has been reported to be induced by the reactivity of antibodies against the neuronal cell surface and caudate-putamen. The activation of NMDAR allows Ca^2+^ and Na^+^ influx into cells, leading to the activation of CaMKII [131]. The activation of CaMKII can mediate inflammatory responses through the ERK/p65/STAT3 or Drp1/ROS/NF-κB pathways, and can also affect dopamine release through the regulation of tyrosine hydroxylase. The CaMKII/ERK/p65/STAT3 signaling pathway is closely associated with inflammation and induces neurotoxicity in dopaminergic neuronal cells [132]. The CaMKII/Drp1/ROS/NF-κB pathway also activates microglia towards pro-inflammatory M1 polarization after stimulation with LPS [116]. CaMKII activation leads to increased tyrosine hydroxylase levels and subsequent dopamine release. CaMKII also regulates the excitability of NMDAR via Glu transmission [133]. Eventually, antibody-mediated CaMKII activation may result in movement disorders, such as TS [109, 134].

#### JAK2/STAT3 pathway

JAK2/STAT3 is considered one of the most important inflammatory pathways that induces the expression of inflammation-related genes. The JAK2/STAT3 pathway is activated by inflammatory factors produced in response to pathogen stimulation. Activation of the JAK2/STAT3 pathway may, in turn, regulate the release of inflammatory factors and interact with downstream transcription factors, such as NF-κB p65, to modulate the inflammatory response. IL-1β, TNF-α, and IL-6 produced in an inflammatory surrounding may activate JAK/STAT signaling, which in turn, can regulate the release of a number of inflammatory cytokines, which may cause neurological damage [118, 135, 136]. Among the JAK2/STAT3 pathway members, STAT3 (signal transducer and activator of transcription 3), a key transcription factor regulating inflammation, can lead to elevated levels of inflammatory cytokines in the brain [119, 137]. LPS can lead to STAT3 phosphorylation [118, 138, 139], after which, STAT3 translocates to the nucleus and acts as a transcription factor, inducing the expression of inflammatory genes. The phosphorylation of STAT may cause the phosphorylation of Janus kinase, and regulate the inflammatory response by interacting with other transcription factors, such as NF-κB p65. LPS induces microglial activation through the JAK2/STAT3 pathway, which regulates the release of inflammatory cytokines [140]. Subsequently, the release of neurotransmitters was regulated, which may lead to the onset of TS [117, 141, 142].

#### NF-κB pathway

Microglia can be activated by LPS through the NF-κB signaling pathway, leading to neuroinflammation [120]. Many pathways, such as the PI3K/Akt, TLR/NLRP3, TLR/MyD88, BDNF/TrkB/MyD88, EGF/EGFR, and Nrf-2/HO-1 pathways, have been shown to regulate NF-κB. PI3K/Akt regulates the NF-κB pathway through the phosphorylation of Akt [121, 122]. Activation of the TLR/NLRP3/NF-κB pathway has been reported to induce inflammation in rat models [123, 143, 144]. The TLR/MyD88/NF-κB pathway has also been shown to be involved in the pathogenesis of TS [123]. Brain-derived neurotrophic factor (BDNF) combined (Trk) receptor, BDNF-tropomyosin-receptor kinase B (TrkB) signaling pathway plays a crucial role in the development of TS by activating the MyD88/NF-κB pathway to regulate the inflammatory response [124, 125, 145]. Inhibition of the Nrf-2/HO‐1 pathway can also lead to activation of the NF‐кB pathway [126]. Inhibition of the EGF/EGFR pathway may activate the NF-κB pathway by inhibiting the Nrf-2/HO-1 pathway, which is involved in inflammation and oxidative stress regulation [127].

#### Other pathways related to TS

The NMDAR/MAPK/CREB pathway plays an important role in the development of TS. Mitogen-activated protein kinase (MAPK) is reported to play important roles in the release of inflammatory cytokines. MAPKs, including c-Jun terminal kinase (JNK), extracellular signal-regulated protein kinase (ERK) and p38, regulate the expression of inflammatory genes [121, 146]. Glu, the release of which is mediated by NMDA receptors, is associated with tic syndromes [147]. Hence, the NMDA-MAPK pathway may lead to TS through the release of inflammatory cytokines and Glu [128]. The PI3K/AKT/mTOR pathway may also be involved in TS pathogenesis [129]. Dopamine release and neuronal growth are regulated by mTOR signaling. The absence of mTOR in the ventral tegmental area alters the balance of neurotransmitters and reduces dopamine levels [148]. In one study, increased levels of FLT3 were observed in patients with TS [149]. Single nucleotide polymorphisms (SNPs) located in the receptor tyrosine kinase gene *FLT3* have been found to activate the PI3K/AKT/mTOR pathway [150].

## Conclusions

**Fig. 2:**
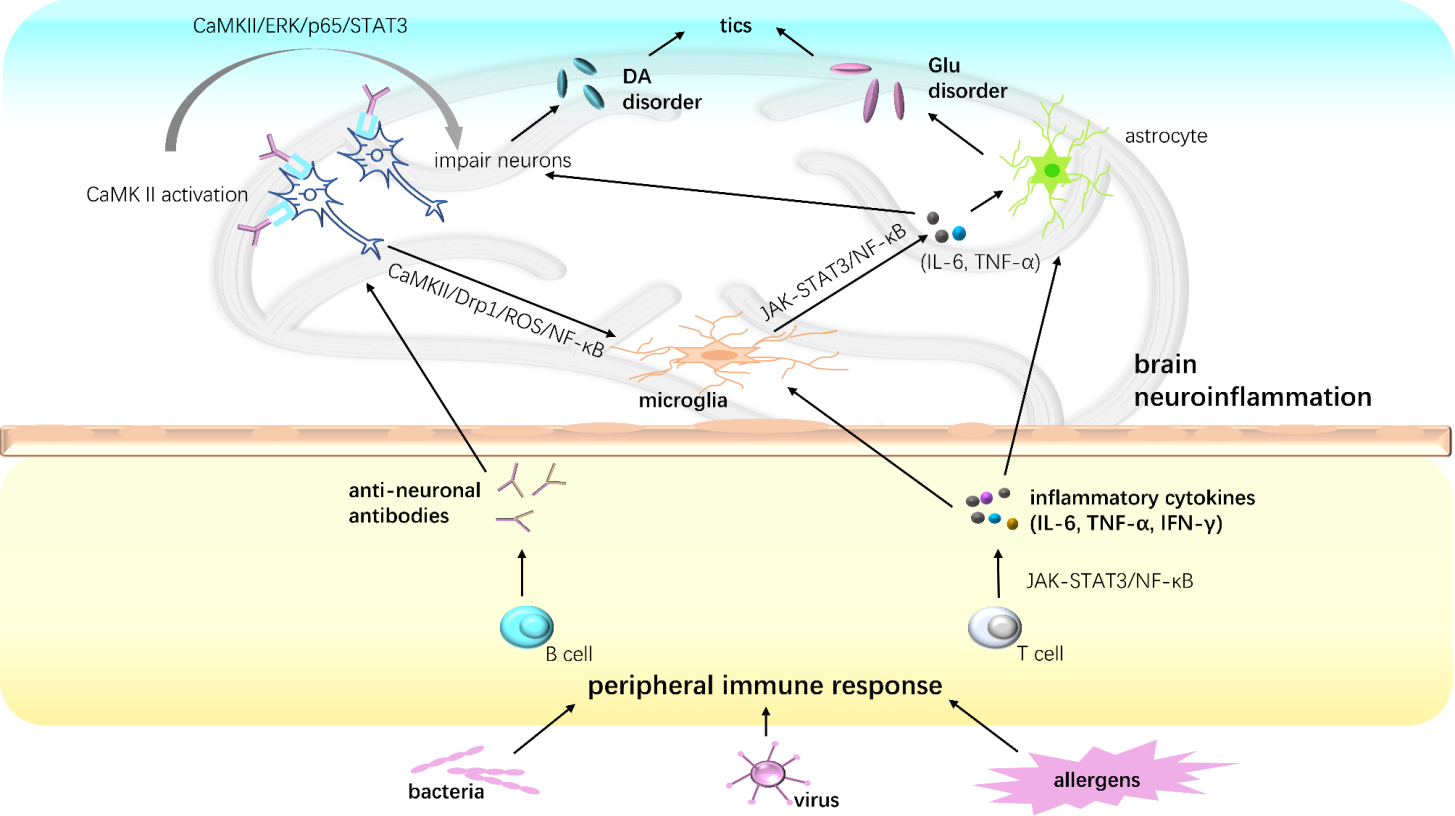
Summary of the possible mechanism leading to Tourette syndrome (TS) associated with the immune response/inflammation. Bacterial infection leads to over-activation of the peripheral immune response, producing a large number of inflammatory cytokines, which diffuse into the brain across the BBB and lead to neuroinflammation. Virus or allergens cloud also mediate the release of inflammatory cytokines. Excessive inflammatory cytokines can also lead to microglia M1 hyperpolarization via the JAK2/STAT3 and NF-κB pathways, which may impair striatal dopaminergic neurons, causing over-release of dopamine (DA), resulting in tics. Anti-neuronal antibodies also play an important role in the pathogenesis of TS mediated by infection. Anti-neuronal antibodies produced after bacterial infection, particularly streptococcus infection, interact with neuronal surface antigens, which activate microglia via the CAMK II pathway, leading to impaired dopaminergic neurons, ultimately, leading to tic syndrome

TS is a neuropsychiatric disorder associated with inflammation-mediated immune response. This article describes the pathogenesis of TS associated with immune responses caused by infection or allergy. Previously, it was found that abnormalities in the CSTC circuit lead to the onset of tics, which are mainly associated with an imbalance in neurotransmitters. Genetic and environmental factors are also involved in the pathogenesis of TS. Inflammation-mediated immune responses can also cause tics, as has been confirmed in numerous clinical and animal studies. Triggers that contribute to the development of tics via inflammatory responses include viral and bacterial infections and allergic reactions. Based on the findings of previous studies, we created a diagram to summarize the underlying mechanism of TS associated with inflammation (Fig. 2). The neurotransmitter imbalance in TS, mediated by neuroinflammation, is a research hotspot involving dopamine and Glu. Tic disorders induced by infection or allergic reactions are commonly observed in clinical practice. Large-sample randomized controlled trials or cohort studies should be conducted to further demonstrate the impact of inflammation-related factors on the onset of tics, which will provide the foundation for exploring novel therapeutic approaches to TS.

## Electronic supplementary material

Below is the link to the electronic supplementary material.

Supplementary Material 1

## Data Availability

Not applicable.
