# Supplementary material for: Neuroinflammation and pathways that contribute to tourette syndrome
Source: Ital J Pediatr. 2025 Feb 28;51:63. doi: 10.1186/s13052-025-01874-3 (PMC11871796; doi:10.1186/s13052-025-01874-3)
Supplement: Supplementary file 1 — Supplementary Material 1 [file 13052_2025_1874_MOESM1_ESM.docx]

Revision Details

| Revision Rationale | Original Content | Revised Content | Revised Pages |
| --- | --- | --- | --- |
| From Reviewer 4:  It would be appropriate to clearly indicate what kind of review this is and to include a paragraph about the research strategy carried out. | / | This article provides a narrative review focusing on inflammation-related factors contributing to the occurrence of TS and the mechanisms by which immune-inflammatory pathways mediate tic onset. A systematic literature search was conducted in databases such as PubMed and Web of Science, including studies from the past two decades on the relationship between TS and immune-inflammatory pathways. The following keywords were used: 'Tourette syndrome', 'inflammation', 'immune', 'microglia', 'neural-immune crosstalk', 'anti-neuronal antibodies', and 'infection'. This review aims to integrate the current evidence on the immune-inflammatory mechanisms underlying TS pathogenesis. | page 2 |
